# Measuring Rigidity During Deep Brain Stimulation Surgery: Evidence of Clinical Benefits in Patients With Advanced Idiopathic Parkinson’s Disease

**DOI:** 10.7759/cureus.89881

**Published:** 2025-08-12

**Authors:** Elodie Lopes, Vânia Almeida, Leonor Dias, Maria J Rosas, Rui Vaz, João P Cunha

**Affiliations:** 1 Faculty of Engineering, University of Porto, Porto, PRT; 2 Engineering, Institute for Systems and Computer Engineering, Technology and Science (INESC TEC), Porto, PRT; 3 Faculty of Medicine, University of Porto, Porto, PRT; 4 Department of Neurology and Movement Disorders and Functional Surgery Unit, Centro Hospitalar Universitário São João, Porto, PRT; 5 Faculty of Medicine, Department of Clinical Neuroscience and Mental Health, University of Porto, Porto, PRT; 6 Clinical Neuroscience Center, Hospital Companhia União Fabril (CUF), Porto, PRT

**Keywords:** clinical efficacy, deep brain stimulation, ihandu system, parkinson's disease, rigidity quantification

## Abstract

Introduction: Wrist rigidity, conventionally qualitatively assessed by clinicians, is an important feature for the success of the deep brain stimulation of subthalamic nucleus (STN-DBS) surgery in patients with Parkinson’s disease (PD). To tackle this subjectivity, our group designed and implemented a wearable device with an embedded sensor, the *iHandU* system (inSignals Neurotech), to quantify the rigidity in real time, supporting the clinical decision-making during STN-DBS.

Methodology: This study aimed to evaluate the clinical benefit of using the *iHandU* device for real-time rigidity quantification during STN-DBS surgery in patients with advanced idiopathic PD. For this purpose, we conducted a retrospective cohort study involving 81 patients with advanced PD undergoing STN-DBS in Movement Diseases and Functional Surgery Unit of the Centro Hospitalar e Universitário São João, Porto, Portugal,, comparing 40 patients assessed intraoperatively with the *iHandU* device to 41 patients assessed by manual methods. Clinical outcomes (motor scores, stimulation parameters, and medication dosage) were compared at multiple time points over a 12-month follow-up.

Results: We found that the *iHandU* group showed significantly higher improvement in motor symptoms with reduced ON/OFF fluctuations and dyskinesias. These gains were obtained with less stimulation current, which reduced the occurrence of side effects, such as speech impairment. Moreover, the *iHandU* group did not show any significantly worse parameters, although sleep disturbances were more frequent (but not statistically significant) than in the control group.

Conclusions: The use of *iHandU* device during STN-DBS was associated with better clinical benefits, namely, by providing improved motor benefits, fewer dyskinesias, and fewer cases of speech disturbance, among other less significant benefits. The use of PD symptom measurement instead of qualitative assessment is probably associated with better clinical outcomes and should be used more frequently in symptom evaluation.

## Introduction

Parkinson’s disease (PD) is a neurodegenerative disorder caused by a loss of dopamine in the substantia nigra. Since dopamine inhibits the propagation of excitatory signals from the nigrostriatal pathway to the motor cortex, its depletion causes the motor pathway to continue to be activated. As a result, bradykinesia, resting tremor, postural instability, and rigidity begin to manifest as PD cardinal symptoms [1].

Since rigidity is present in 90-99 % of PD patients, clinicians usually impose a passive flexion to a patient’s joint and attribute it a subjective rigidity improvement score (ranging from 0 % to 80% [1]) for each tested position and stimulation parameters, translating the rigidity severity change in response to a therapeutic approach and select the best setup for each patient. The wrist rigidity, for instance, is a reliable feature that allows the detection of cogwheel rigidity, which is a PD-typical muscular stiffness [1]. A second neurologist may perform an intra-operative blind test to confirm the first evaluation, over the three best electrode positions to reach a clinical consensus for implantation. However, this assessment is subjective and depends on the perception and experience of each neurologist. This highlights the need for the creation of quantitative methods for rigidity assessment in this neurosurgery scenario.

To tackle this subjectivity, our group has been working for almost 10 years on a wearable device to quantitatively assess the wrist rigidity changes during the DBS procedure, supporting physicians in decision-making when locating electrodes and setting the stimulation parameters. This system, named *iHandU* (inSignals Neurotech), comprises a precise inertial motion sensor embedded in a textile band to be worn on the patient’s hand, which communicates via Bluetooth to a smartphone where data analytics is performed in near-real time. It measures motion signals that are analyzed to extract key features used to model rigidity and obtain an improvement measure. Its usage is very simple and does not change the routine workflow of the surgery [2]. The rigidity improvement is computed by deriving features from angular velocity patterns during wrist flexion manoeuvres and using a polynomial mathematical model to classify these patterns into levels of improvement. For the last several years the device has been evaluated on three datasets where several data analysis and classification models were tested on an overall of 1,494 rigidity measurements collected during DBS surgeries from 118 patients, showing an average accuracy of 80% (min. 77%; max. 82%) against the blind agreement of two experts [3-5].

Since the first version, developed in 2015 and described in [2], the *iHandU* system has undergone several developments in terms of software, hardware, and classification model, to improve the system performance, as well as making it more suitable for intraoperative conditions and more comfortable for both patients and clinicians. This gave rise to three new versions over the years: the *iHandU* 2.0, described in [3], the *iHandU* 3.0, described in [4], and our more recent version, the *iHandU* 4.0, which was developed to improve system comfort at the patient and clinician levels, as well as to enable faster signal processing and an easier methodology to record surgery-related information [5]. Currently, a new version has been developed to tackle the limitations found in the system. For this purpose, accelerometers, magnetometers, and force sensors were added to the system, and new classification models have been developed. A multi-center clinical study is being performed involving DBS centers from Portugal, the Netherlands, Germany, and Spain to show the reproducibility of the device and prepare for its future entrance into the medical device market.

In this study, we intend to assess the clinical benefit of using the *iHandU* device during DBS in patients with PD. Since no studies were found in the literature making direct comparisons between similar devices, a literature survey was performed to identify potential predictive DBS-clinical efficacy features. For this purpose, we implement the PICO model (P-Population/Problem; I-Intervention; C-Comparator, O-Outcome) on the PubMed platform [6], and we found seven main groups of relevant predictive DBS-clinical efficacy features, designated as demographic, clinical, medication-related, surgery-related, rigidity, adverse effects, and follow-up [7-20].

Demographic features include gender [7,8], age at diagnosis [9], age at surgery [8,10,11], and duration of disease [9]. Clinical features are related to conventional PD assessment scales and the mortality rate [7,8,18]. Commonly used PD assessment scales include the Hoehn and Yahr (H&Y) staging, measuring the disease severity [7-20]; the MDS-Unified Parkinson’s Disease Rating Scale (MDS-UPDRS), measuring motor impairment and sub-divided into MDS-UPDRS III [7-20] and MDS-UPDRS IV [12,13]; and the Parkinson Disease Questionnaire-39 (PQD-39 Score) [14-17], measuring the quality of life. Medication-related features include the total daily dose (mg) of levodopa that the patient was taking until the day before surgery and daily doses that the patient started taking three months and 12 months after surgery [7,8,14]. Surgery-related features include the stimulation parameters, such as electrode location, depth, and stimulation current [13,16,19], whereas rigidity features show the percentage of rigidity improvement [2-5]. Adverse effects can be due to surgery [9,14], stimulation [9,14], hardware [9,14,15], or falls [9,14,15], and follow-up features include the number of hospital visits due to emergency episodes [20] or the need to adjust stimulation parameters [7].

The primary objective of this study was to evaluate the clinical benefit of using the *iHandU* wearable system for intraoperative quantitative assessment of wrist rigidity during subthalamic nucleus deep brain stimulation (STN-DBS) surgery in patients with advanced idiopathic PD. Specifically, we aimed to compare clinical outcomes between patients assessed using the *iHandU* system and those assessed using standard conventional methods. Secondary objectives included evaluating differences in motor symptoms, medication dosage, stimulation parameters, and adverse effects.

## Materials and methods

In this work, we present an observational, analytical retrospective cohort study in which 22 predictive DBS-clinical efficacy features were extracted from 81 patients with idiopathic PD who maintained bilateral STN-DBS stimulation for at least 12 months. These patients were followed up at the Movement Diseases and Functional Surgery Unit of the Centro Hospitalar e Universitário São João, Porto, Portugal, a center of excellence that has regularly performed this surgery since 2003. To assess the eventual clinical benefit of the *iHandU* system, we compared clinical efficacy features from 40 patients in whom the *iHandU* device was used during DBS surgery and 41 patients in whom the device was not used. This study was approved by the Ethics Committee of the Centro Hospitalar e Universitário São João (project name ID: WearPark 2.0) in January 2018.

Dataset

We selected 81 from a total of 108 patients who underwent bilateral STN-DBS surgery in the DBS center and had computerized medical records available. Inclusion criteria included advanced idiopathic PD patients who underwent bilateral STN-DBS for the first time and maintained active stimulation for at least 12 months. Exclusion criteria included patients implanted in the globus pallidus (GPi) and patients who had already undergone a previous STN-DBS and unilateral DBS cases. The dataset was then divided into two subgroups: the control group (patients for whom the *iHandU* device was not used) and the *iHandU* group (patients for whom the *iHandU* device was used during surgery). Table 1 shows the dataset characterization. The control group included 41 patients operated during the period from 2013 to 2014, and the *iHandU* group 40 patients operated between 2018 and 2020 (used the more recent system). Note that this DBS center has been performing DBS surgery since 2003. Between the period 2003 and 2013, for instance, around 150 surgeries were performed. Nevertheless, the surgery procedure and mostly the clinical team remained the same over the years. The Appendix provides an overview of the DBS systems employed throughout the study period. Patients operated on after 2020 were not included due to the pandemic situation that imposed limitations on the presence of engineers during surgery.

**Table 1:** Dataset characterization in terms of gender, age distribution, and disease duration of the 81 patients involved in the study.

	Control group (n = 41)	iHandU group (n = 40)
Number of females	15	21
Number of males	26	19
Age at surgery (y)	58.6 ± 7.7	61.8 ± 8.2
Age at diagnosis (y)	47.1 ± 8.0	50.5 ± 7.8
Disease duration (y)	12.2 ± 5.8	12.3 ± 4.5

Surgical procedure and intraoperative assessment

The stereotactic planning was performed on the day of surgery using a preoperative 1.5T MRI merged with a stereotactic CT scan to localize the anatomically defined target [21]. The final target position was determined through direct visualization of the STN in the MRI. The surgery was conducted with the patient in the supine position, with a 30º head flexion, under local anesthesia [21].

The standard protocol involved three trajectories for intraoperative microelectrode recording (MER) to identify the neurophysiological signature of the STN: central (target), anterior, and lateral [22]. Typically, MER was initiated 5 mm above the planned target, advanced in 0.5 mm increments, and extended 3-5 mm below the target until the substantia nigra was detected [22,23]. During this step, two experienced neurologists performed visual and auditory analyses of the single-unit recordings captured by high-impedance electrodes [22].

To determine the optimal electrode placement, intraoperative microstimulation was performed at two different depths using each microelectrode, while assessing clinical benefit and adverse effects [22]. To evaluate rigidity, neurologists imposed wrist flexions on the patient’s hand while varying the voltage and placement, with the stimulation frequency set at 130 Hz. The optimal setting was agreed upon by two experienced physicians. For patients in the *iHandU* group, the signal classification presented by the *iHandU* system was compared to the evaluations of the two experts.

Post-surgery, a CT scan was performed and merged with the preoperative MRI to verify the final position of the electrodes and to rule out hemorrhage or ischemia [22].

*iHandU* device and signal processing

The *iHandU* device comprises an inertial motion sensor embedded in a textile band placed on the patient’s hand. During intraoperative wrist flexion maneuvers, angular velocity data is collected and transmitted in real-time to a processing unit. Signal processing includes feature extraction (e.g., angular velocity peaks, angular velocity average values) and classification using a polynomial model trained on labelled rigidity improvement levels. Further details on algorithm development and validation can be found in [2-5].

Predictive DBS-clinical efficacy features and data collection

For each dataset group, we retrieved and compared a total of 24 predicted DBS-clinical efficacy features (Table 2) based on the above-mentioned literature review. Data were gathered from each patient’s computerized medical record electronically available through the sClinico-SPMS Min. Health Software and some features had to be obtained from the related patient records available at the hospital.

**Table 2:** Selected predictive DBS-clinical efficacy features. Abbreviations: MDS-UPDRS III Med OFF/Stim OFF: MDS-UPDRS III prior the stimulation and levodopa test; MDS- UPDRS III Med ON/Stim OFF and MDS-UPDR IV Med ON/Stim OFF: MDS-UPDRS III and MDS-UPDRS IV following the acute levodopa test and before the DBS procedure; MDS-UPDRS III Med ON/Stim ON 3M and 12M: MDS-UPDRS III 3 and 12 months, respectively, after DBS surgery; MDS-UPDRS IV Med ON/Stim ON: MDS-UPDRS IV one week after DBS surgery; Levodopa Stim OFF: total daily levodopa dose that the patient took until the day before surgery; Levodopa Stim ON 3M and 12M: total daily levodopa dose took by the patient three and 12 months after surgery.

Predictive DBS-clinical efficacy features	Description
Demographic features	Gender; age at diagnosis (years); age at surgery (years); disease duration (years); comorbidities
Clinical features	MDS-UPDRS III Med OFF/Stim OFF; MDS-UPDRS III Med ON/Stim OFF; MDS-UPDRS III Med ON/Stim ON 3M; MDS-UPDRS III Med ON/Stim ON 12M; MDS-UPDRS IV Med OFF/Stim OFF; MDS-UPDRS IV; Med ON/Stim ON
Medication-related features	Levodopa Stim OFF (mg); levodopa Stim ON 3M (mg); levodopa Stim ON 12M (mg)
Surgery-related features	Stimulation location; depth (mm); stimulation current amplitude (mA)
Rigidity features	Rigidity improvement (%)
Adverse effects features	Surgery-related effects; stimulation-related effects; hardware-related effects; falls
Follow-up features	Urgency episodes; stimulation parameter adjustments

**Figure 1:**
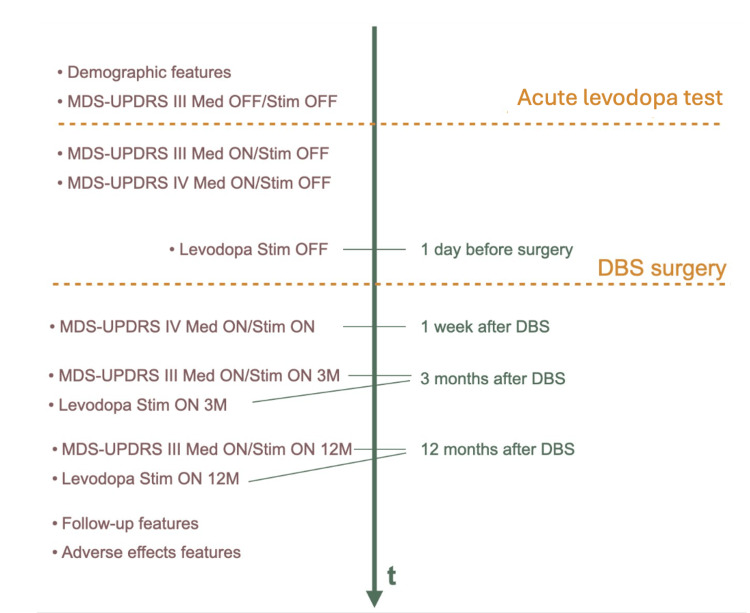
Timeline of data collection and respective efficacy features time of collection.

In Fig. 1, the evaluation moments of each clinical efficacy feature can be depicted. Before the stimulation and levodopa test, the demographic features (gender, age at diagnosis, age at surgery, and comorbidities) and MDS-UPDRS III subscale score (MDS-UPDRS III Med OFF/Stim OFF) were gathered. Osteoarticular pathology, cardiovascular pathology, mental pathology, neurological pathology (other than PD), and other pathologies (for those that did not fit into any of the above) were the categories used to classify comorbidities.

Then, following the acute levodopa and prior to the DBS procedure, we gathered MDS-UPDRS III Med ON/Stim OFF, MDS-UPDRS IV Med ON/Stim OFF, and the levodopa Stim OFF data (total daily levodopa dose that the patient took until the day before surgery). The PQD-39 score and HY staging data were not gathered since they were not consistently evaluated at the study site for all patients.

Surgery-related (stimulation location, depth, and current amplitude) and rigidity-related (rigidity improvement) features were gathered at the time of surgery. The improvement in rigidity was qualitatively evaluated based on the consensus of two neurologists using wrist rigidity manoeuvres. The best electrode location and stimulation parameters (current, frequency, depth, stereotactic location) were selected based on the neurologists’ best joint agreement. For the *iHandU* Group, neurologists performed the same above-mentioned procedure. After a first agreement, they asked for the *iHandU* quantitative evaluation output and considered the calculated percentage of improvement from the system, eventually adjusting their agreement with further maneuvres and discussion.

Finally, following surgery, we gathered MDS-UPDRS IV Med ON/Stim ON (one week after DBS surgery), MDS-UPDRS III Med ON/Stim ON 3M and levodopa Stim ON 3M (motor score and patient’s total daily levodopa dose taken three months after surgery), and MDS-UPDRS III Med ON/Stim ON 12M and levodopa Stim ON 12M (motor score and patient’s total daily levodopa dose took by the patient 12 months after surgery). Adverse effects and follow-up episodes, such as speech and gait disturbances, the number of hospital admissions resulting from emergency events or changes to the stimulation parameters, were also recorded during the follow-up period.

Data analysis

This study aimed to understand whether the *iHandU* group presented any clinical benefit over the control group. Beneficial effects can be translated into a greater reduction in MDS-UPDRS III and IV scores, fewer adverse effects, lower daily levodopa doses, fewer adjustments of stimulation parameters, lower stimulation current, and fewer stimulation-related emergency episodes. For this purpose, we performed a statistical data analysis using the IBM SPSS Statistics for Windows, Version 26.0 (released 2018, IBM Corp., Armonk, NY). Absolute and relative frequencies were used to characterize categorical features, whereas the mean and standard deviation were used for continuous variables. We then used non-parametric tests (Table 3) due to the non-normality of most of the features used (tested with the Shapiro-Wilk Test [24]). Differences were considered statistically significant when the significance value was less than 0.05 (p < 0.05). Data with incomplete entries for critical clinical or surgical variables were excluded from statistical analysis on a case-by-case basis. No imputation methods were applied.

**Table 3:** Statistical tests used to compare features extracted from control and iHandU groups.

Non-parametric test	Features
Mann-Whitney test	iHandU group vs. control group (continuous variables)
Wilcoxon test	MDS-UPDRS IV Med On/Stim OFF vs. MDS-UPDRS IV Med ON/Stim ON
ANOVA Friedman test	MDS-UPDRS III Med ON/Stim OFF vs. MDS-UPDRS III Med ON/Stim ON 3M and 12 M Levodopa Sim OFF vs. Levodopa Stim ON 3M and 12M
Fisher's exact test	iHandU group vs. control group (categorical variables)

## Results

**Figure 2:**
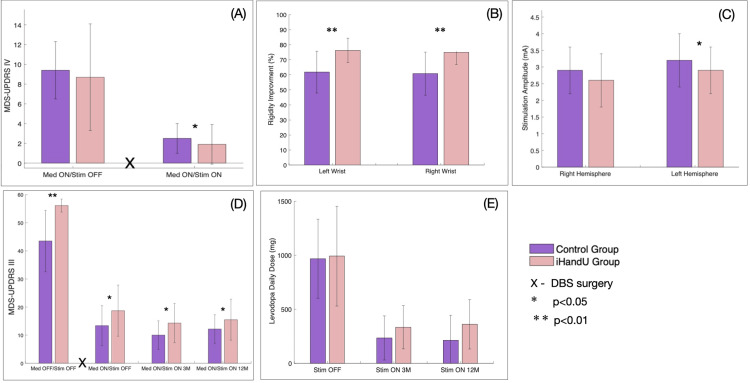
Comparative clinical features changes (average ± std.) between the control and iHandU groups: (A) MDS-UPDRS IV scores before and one week after surgery (statistical analyses were performed using the Wilcoxon test with a threshold value for significance of p < 0.05); (B) agreement on rigidity improvement with DBS by two specialists (statistical analyses were performed using the Mann-Whitney test with a threshold value for significance of p < 0.05); (C) stimulation current amplitude (statistical analyses were performed using the Mann-Whitney test with a threshold value for significance of p < 0.05); (D) MDS-UPDRS III evolution (statistical analyses were performed using the ANOVA test with a threshold value for significance of p < 0.05); and (E) daily medication dosage at the different evaluation follow-up moments (statistical analyses were performed using the ANOVA test with a threshold value for significance of p < 0.05).

**Figure 3:**
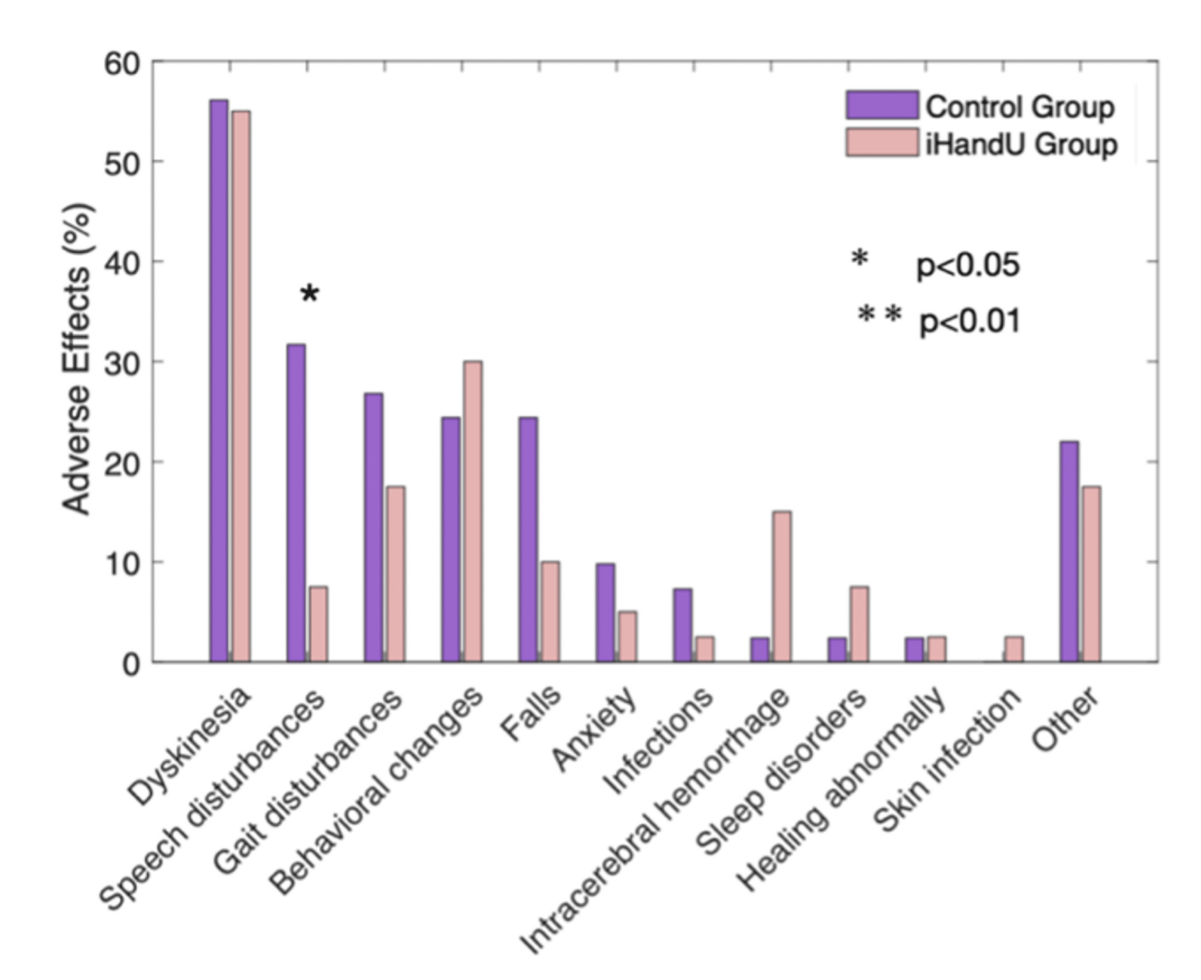
Presence of different adverse effects throughout the 12-month evaluation period for both groups (in %). Statistically significant changes were found in speech disturbances. In most of the other analyzed adverse effects, the iHandU group presented lower incidence, although not statistically significant (statistical analyses were performed using the Fisher's exact test with a threshold value for significance of p < 0.05).

Several clinical, medication-related, surgery-related, rigidity-related, and adverse effects features were shown to have a statistically significant difference between the control and *iHandU* groups (Figs. 2, 3).

## Discussion

The *iHandU* group outperformed the group control in terms of improvement of motor fluctuations following surgery, as evidenced by significantly significant lower UPDRS IV scores (p < 0.05) (Fig. 2A), better rigidity improvement (p < 0.01) (Fig. 2B), based on the consensus of two experts, and attaining these improvements with lower stimulation current (p < 0.05) (Fig. 2C).

Prior to surgery, MDS-UPDRS III scores (Fig. 2D) were considerably higher in the *iHandU* group when compared to the control group (p < 0.01). These scores continued to follow the same trend both three and 12 months following surgery (MDS-UPDRS III Med ON/Stim ON 3M and 12M) (p < 0.05), with both groups experiencing a remarkable overall decline in follow-up after surgery, as expected It is worth mentioning that the statistical difference between groups were much higher before (p < 0.01) than after surgery (p < 0.05). This indicates that the severity ”difference” between the groups was reduced in relation to expected if no usage of the *iHandU* system was present. Medication dosages were also significantly lowered after surgery (Fig. 2E) in both groups, being higher for the *iHandU* group, probably associated with the higher MDS-UPDRS scores in these patients before and after surgery, indicating that patients of the *iHandU* group presented more severe motor impairment before surgery.

In addition, the *iHandU* group experienced considerably fewer speech disturbances (p < 0.05) (Fig. 3) and tended to experience fewer adverse effects, such as gait disturbances, falls, and anxiety episodes in the 12 months following DBS surgery. By contrast, this group had slightly more behavioural alterations and a tendency toward more sleep disturbances.

Regarding the demographic features (gender, age at diagnosis, age at surgery, disease duration, and comorbidities), there were no statistical differences between the two groups. The mean age of the patient at surgery (59.1 ± 2.9 years) and the disease duration (12.2 ± 2.1 years) agreed with a systematic review performed by Lachenmayer et al. [13]. The most common comorbidities were the osteoarticular pathology (26.8% in the control grouup and 37.5% in the *iHandU* group), cardiovascular (26.8% in the control group and 42.5% in the *iHandU* group), psychiatric (29.3% in the control group and 20.0% in the *iHandU* group), and neurological pathology (4.9% in the control group and 5% in the *iHandU* group). The most frequent location for the exploration of the DBS electrode was the central area in both groups and for both hemispheres, located deeper for the *iHandU* group than for the control group, also in both hemispheres. These results point to a very good targeting direction of the electrode trajectory by the multidisciplinary surgery team but to a slightly deeper positioning for the *iHandU* group than the group control, probably resulting in less “capsule effect”, contributing to the statistically significant lower speech impairment (p < 0.05) found in this group.

An important note is that the above-mentioned outperformance of several clinical efficiency features of the *iHandU* group was obtained recurring to less stimulation current (p < 0.05) that is known to pose a lower probability of occurrence of adverse side effects during follow-up for these patients [25] and provide longer stimulator’s battery life, thus providing long-term clinical benefits for patients and reducing the overall costs of treatment.

In addition to the significant clinical benefits observed in the *iHandU* group, some non-significant or counterintuitive trends were noted, such as increased reports of sleep disturbances and behavioral alterations. These may be due to individual variability, postoperative stimulation settings, or reporting bias and warrant further exploration in larger, prospective studies. In addition, trends showing reduced anxiety and infection in the *iHandU* group were not statistically significant and may reflect random variation or differences in perioperative care practices. These findings underscore the need for cautious interpretation and more controlled studies to understand their clinical relevance.

Despite the positive and novel outcomes of this study, we draw attention to some possible drawbacks: we were unable to get pre- and postoperative data from the PDQ-39 scale and H&Y staging since they were not consistently assessed at the study site. However, the state of the art highlights the importance of this scale in the assessment of the clinical efficacy of DBS, and we should consistently use it in the future. It was also not possible to compare the mean duration of the STN-DBS surgery procedure for both groups, to assess whether the *iHandU* device can be advantageous in supporting medical decisions while reducing or enlarging surgery duration. Nevertheless, we have minimized the time of *iHandU* measure to <5 seconds for each position/stimulation parameter trial, and its setup on the hand of the patients does not take more than one minute. Regarding medication-related features, we could not include other antiparkinsonian drugs due to sample size limitations.

Strengths of this study include its real-world clinical setting, innovative technology application, and a 12-month follow-up period. However, several limitations must be acknowledged: First, this was a retrospective, non-randomized observational study, and as such, it is subject to inherent biases such as selection bias and confounding. The absence of randomization and blinding may have influenced both intraoperative decisions and postoperative outcomes. Therefore, these results should be considered exploratory and hypothesis-generating rather than conclusive. Second, this study concerns the absence of systematic recording of surgical duration, which may limit assessments of intraoperative workflow efficiency. Third, the control and *iHandU* groups underwent surgery in different time periods (2013-2014 vs. 2018-2020), which could have introduced variability due to evolution in DBS targeting, equipment, or perioperative care. While the surgical team and core protocol remained stable (both clinical leaders - neurosurgeon and neurologist, and several of the other involved members remained on the team over the years), unmeasured factors may have contributed to observed differences and must be considered when interpreting the results. Fourth, although postoperative imaging was used clinically to confirm electrode placement, we did not perform a detailed analysis of final lead localization, which is known to influence DBS efficacy. Fifth, certain outcomes, such as sleep disturbances and behavioral effects, did not reach statistical significance but showed differing trends between groups. These results warrant further investigation in future prospective studies with larger sample sizes and standardized adverse event monitoring.

## Conclusions

The goal of the current study was to determine whether measuring rigidity improvement during STN-DBS surgery with the wearable *iHandU* technology in patients with advanced idiopathic PD would provide any clinically beneficial evidence in a 12-month follow-up. To achieve this, we retrieved and evaluated 24 predictive DBS-clinical efficacy features for 40 patients in whom the *iHandU* device was used during the DBS implantation surgery procedure (*iHandU* group) and 41 patients who did not (control group). We found that the *iHandU* group presented an improvement in ON/OFF fluctuations and dyskinesia motor symptoms (statistically significant lower UPDRS IV scores), reducing them to minimal or no motor complications for these patients (score = 2), and presenting a larger reduction of rigidity based on the agreement of two specialists. Furthermore, these notable clinical gains were made possible with less stimulation current, which reduces the likelihood of future side effects for these patients and provides longer stimulator battery life, thus obtaining evidence of clinical benefits for the patients at lower costs. In addition, these individuals experienced noticeably reduced speech impairment, indicating a possible reduction of the capsule effect when compared with the control group. Nevertheless, other motor symptoms followed the expected improvement pattern (MDS-UPDRS III feature), presenting higher scores before and after the surgery and a corresponding levodopa dosage higher for the *iHandU* group (although not statistically significant).

In conclusion, the use of the *iHandU* system during STN-DBS surgery was associated with improved motor outcomes, fewer stimulation-related side effects, and reduced current amplitude. These findings suggest that quantitative intraoperative rigidity assessment may support more precise electrode placement and optimized stimulation settings. However, due to the study’s retrospective and non-randomized design, these results should be interpreted with caution. Future controlled, prospective studies are needed to confirm these findings across different DBS centers. Such a clinical study is already being prepared in centers from Portugal, the Netherlands, Germany, and Spain to show the reproducibility of these results and prepare for its possible future entrance into the medical device market. In addition, further studies should investigate whether the implementation of this system can contribute to reducing the surgery duration and improving the overall procedural efficiency. These ongoing efforts will be crucial in validating the clinical utility of *iHandU* and accelerating its path toward regulatory approval and widespread adoption in PD treatment.

## Appendices

**Table 4:** Medtronic STN-DBS neurostimulation systems utilized throughout the experimental period of this study at Centro Hospitalar e Universitário de São João The Activa™ PC is a non-rechargeable, dual-channel neurostimulator designed for standard DBS therapy, typically lasting 3–5 years. The Activa™ RC is a rechargeable version with a longer lifespan of up to 15 years, ideal for patients requiring high energy output or long-term therapy. The most advanced of the three, Percept™ PC, is also non-rechargeable but features BrainSense™ technology, enabling real-time sensing of brain signals to support adaptive DBS and more personalized treatment.

Year	STN-DBS System
2013	Activa PC
2014	Activa PC
2015	Activa PC, Activa RC
2016	Activa PC
2017	Activa PC
2018	Activa PC, Activa RC
2019	Activa PC, Activa RC
2020	Activa PC, Activa RC and Percept PC
